# Properties, Engineering Applications, and Mechanisms of Yellow River Silt and Sand in Construction Materials: A Comprehensive Review

**DOI:** 10.3390/ma19163412

**Published:** 2026-08-11

**Authors:** Yufei Chang, Shupeng Xiao, Zhi Zhou, Xiaofei Hu, Yifei Wang, Ziheng Du

**Affiliations:** 1 School of Civil Engineering and Architecture, Anyang Normal University, Anyang 455000, China; hxiaofei@stu.aynu.edu.cn (X.H.); wyf@stu.aynu.edu.cn (Y.W.); dzh@stu.aynu.edu.cn (Z.D.); 2 Henan Engineering Technology Research Center for Digital Intelligent Building and Low Carbon Building Materials, Anyang Normal University, Anyang 455000, China; 3 Network Information Center, Anyang Normal University, Anyang 455000, China; xsp@aynu.edu.cn; 4 School of Civil Engineering and Architecture, Hainan University, Haikou 570228, China; zhizhou@hainanu.edu.cn

**Keywords:** Yellow River sediment, Yellow River silt, Yellow River sand, construction materials, fine aggregate, interfacial transition zone (ITZ), pore evolution, physicochemical mechanisms

## Abstract

**Highlights:**

**What are the main findings?**

- Yellow River sand mainly serves as fine aggregate in concrete, ECC, and cement mortar.
- Yellow River silt mainly acts as a filler or weakly reactive component.
- Effects of Yellow River silt and sand are governed by physical packing and chemical reactivity.

**What are the implications of the main findings?**

- Content ranges depend on material system and target performance.
- Future research should develop predictive models and clarify long-term durability.
- Low-carbon activation remains a key priority for Yellow River silt.
- Regional variability hinders standardized engineering use of Yellow River sediment.

**Abstract:**

Yellow River sediment is a promising resource for use in construction materials. However, the effects of Yellow River silt and sand in different applications remain unclear. This review examines the physicochemical properties of these two forms, their engineering applications, and the underlying mechanisms governing material performance. Yellow River sand is mainly used as a fine aggregate in conventional concrete, engineered cementitious composites (ECC), and cement mortar, whereas Yellow River silt is commonly used in subgrade fills, concretes incorporating multiple solid wastes, and building products. Across these applications, the behavior of Yellow River sediment in construction materials reflects both physical packing and chemical reactivity. Their relative contributions depend on sediment form, material system, processing condition, and sediment content. Chemical reactivity is observed mainly in systems containing Yellow River silt, although the contribution of the silt itself has not been isolated. In conventional concrete, the highest compressive strengths are generally observed when Yellow River sand replaces 10–30% of natural sand, but the exact level depends on mixture design. Regional differences in sediment composition further limit the direct transfer of this range and require evaluation based on target performance. Future research should develop predictive models linking sediment characteristics, content, and target performance, establish low-carbon activation methods, and clarify long-term durability under complex service conditions.

## 1. Introduction

The Yellow River is one of the major rivers in northern China and is known for its high sediment load [1,2]. Long-term erosion and downstream transport have led to the continuous accumulation of fine particles in the middle and lower reaches, forming large deposits that disrupt channel stability and increase maintenance costs [3,4,5]. Nevertheless, the distinctive particle-size distribution, mineralogy, and chemical composition of these sediments indicate their potential for use in construction materials [6,7,8]. With the growing demand for resource recycling and sustainable construction materials, Yellow River sediment has attracted growing interest as a valuable material resource rather than a management burden [9,10].

The terms Yellow River silt and Yellow River sand are widely used in the literature [6,11,12], but their definitions are not fully consistent across studies. In this review, Yellow River silt refers to unscreened natural fine-grained sediment, whereas Yellow River sand refers to the sand fraction obtained through washing and sieving. The distinction therefore reflects material state and processing rather than particle size alone. Despite their different functions in construction, Yellow River silt and Yellow River sand originate from the same sedimentary source and share fundamental characteristics [13,14,15]. A unified analytical perspective makes it possible to distinguish their different engineering roles while clarifying their shared underlying mechanisms.

Although studies on Yellow River silt and sand in construction materials have increased in recent years, most focus on single material systems, such as concrete [16,17,18], cementitious materials [19,20,21], subgrade fills [22], or building products [23,24]. Recent reviews have mainly focused on the activation and performance of river sediments in cementitious materials [25] and the use of dredged sediments in alkali-activated materials [26]. However, the differences between Yellow River silt and Yellow River sand in engineering response, governing mechanism, and application boundary remain insufficiently clarified, particularly in relation to the balance between densification and dilution effects [27,28,29], regional variability [30,31], and long-term microstructural evolution [32].

Accordingly, this review aims to clarify the different roles of Yellow River silt and Yellow River sand in construction materials. Across different construction applications, the review compares the performance of the two sediment forms. Material characteristics, mixture design, processing conditions, and binder environments are considered when explaining the reported differences. The relevant physicochemical mechanisms are further examined with attention to their supporting evidence and applicability limits. The main contribution is an application-oriented synthesis that links each sediment form to its engineering role, main engineering effect, key limiting factor, and dominant mechanism. This review is expected to support the rational use of Yellow River sediment and to provide a reference for other fine-grained sediments with similar physical, mineralogical, or reactivity characteristics.

## 2. Material Properties

### 2.1. Particle Size and Morphology

The natural sediment of the Yellow River exhibits a wide range of particle sizes, primarily consisting of clay, silt, and sand [7,33]. In engineering studies, the names Yellow River silt and Yellow River sand generally reflect the dominant particle fraction in the material rather than a strict separation into two particle size classes [34].

**Figure 1:**
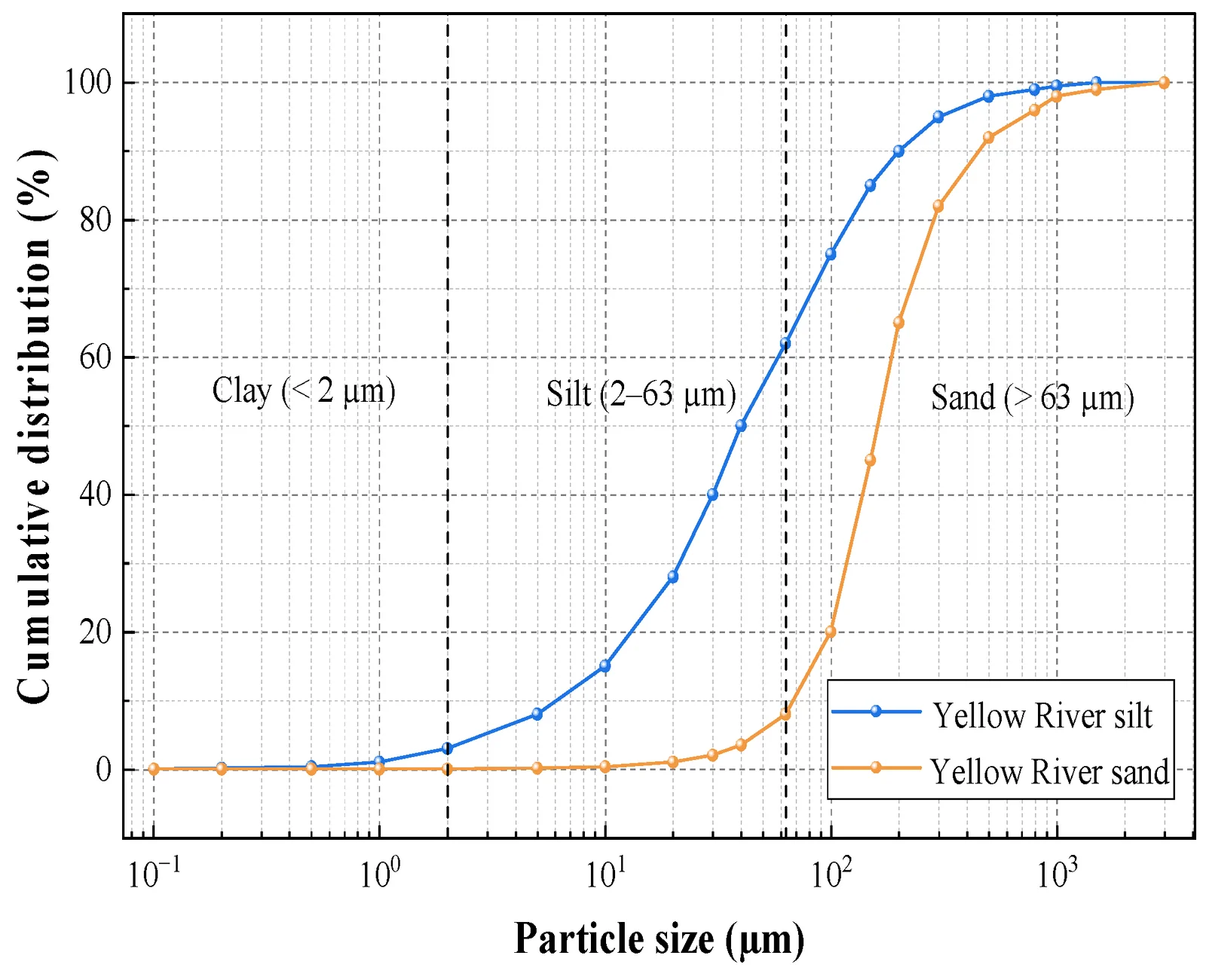
Representative particle-size distributions of Yellow River silt and Yellow River sand [37,38].

As a natural and unscreened sediment, the Yellow River silt is dominated by fine-grained particles. Its particle size typically ranges from 0.005 to 2 mm. This high content of ultra-fine particles creates a large specific surface area. In contrast, Yellow River sand has a coarser gradation, with particle sizes ranging from 0.075 to 2 mm. However, it still contains a higher proportion of fine particles than conventional river sand and has a specific surface area up to 5.77 times that of standard quartz sand [35,36]. Although their particle size ranges overlap, the two sediment forms differ in the relative proportions of fine and coarse particles. Figure 1 presents representative particle-size distributions of Yellow River silt and Yellow River sand [37,38].

Both sediment types have rounded particles resulting from long-term hydrodynamic transport and abrasion [39,40,41]. This morphology minimizes inter-particle friction and improves packing efficiency [42]. For Yellow River silt, these attributes facilitate dense packing within the material and help regulate mixture rheology [40,41]. For Yellow River sand, the rounded grains and stable grading optimize inter-particle contact and the aggregate skeleton structure [43]. These particle characteristics are directly associated with packing behavior and, consequently, with the macroscopic performance of the resulting materials [42,43].

**Figure 2:**
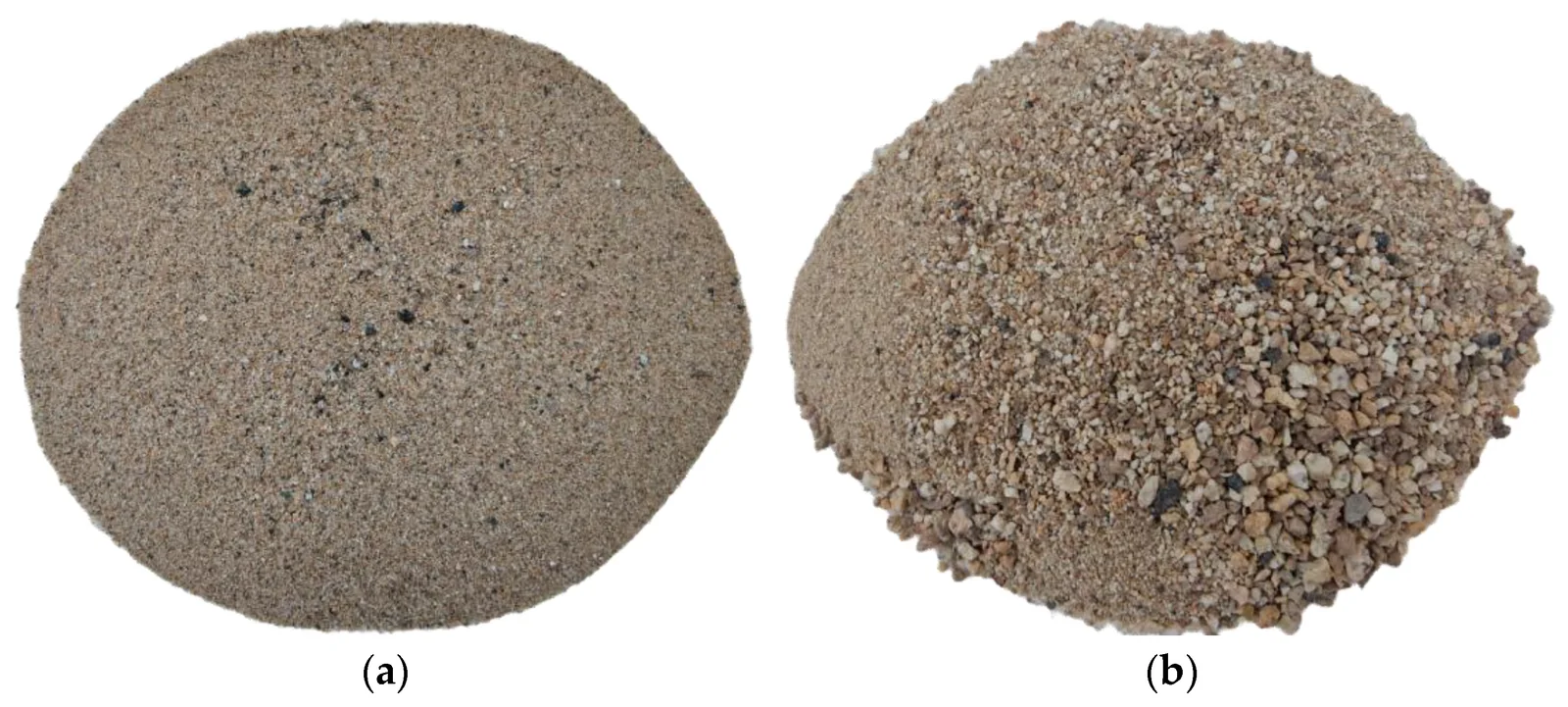
Macroscopic appearance of Yellow River sand and manufactured sand: (**a**) Yellow River sand; (**b**) manufactured sand.

Figure 2 illustrates the macroscopic appearance of Yellow River sand and manufactured sand. Yellow River silt is excluded from this visual comparison because its ultra-fine nature makes macroscopic features less discernible. As shown in Figure 2, Yellow River sand exhibits a markedly finer and more uniform appearance than the angular grains of manufactured sand. This contrast visually supports the high roundness and stable gradation resulting from natural transport processes [39,43].

### 2.2. Mineral and Chemical Composition

Yellow River sediment is mainly composed of non-clay minerals such as quartz and feldspar. Alongside these primary components, it contains some clay phases, specifically illite, montmorillonite, and chlorite [44,45]. These constituents exhibit strong adsorption and ion-exchange capacities, thereby influencing water absorption and admixture compatibility in cementitious mixtures [35,46,47].

The content of clay minerals differs significantly between the Yellow River silt and the Yellow River sand. Yellow River silt contains a much higher clay-mineral content [48]. In low water-to-cement ratio concrete, these clay minerals can adsorb water-reducing agents, leading to a decrease in mixture workability [49,50]. Conversely, in washed Yellow River sand, clay primarily exists as a minor associated impurity, and its adverse effect on admixture adsorption can usually be reduced by moderate washing [51]. Saturated surface-dry water absorption values of 1.1% [52] and 1.9% [53] have been reported for Yellow River sand. No corresponding value has been reported for Yellow River silt in the available literature.

In terms of chemical composition, Yellow River sediment is dominated by SiO_2_ and Al_2_O_3_, with smaller amounts of CaO, Fe_2_O_3_, and K_2_O [7,25]. The sediment contains 0.4–0.8% organic matter and is slightly alkaline, with a pH of 7.5–8.5 [54,55].

### 2.3. Regional Differences

**Figure 3:**
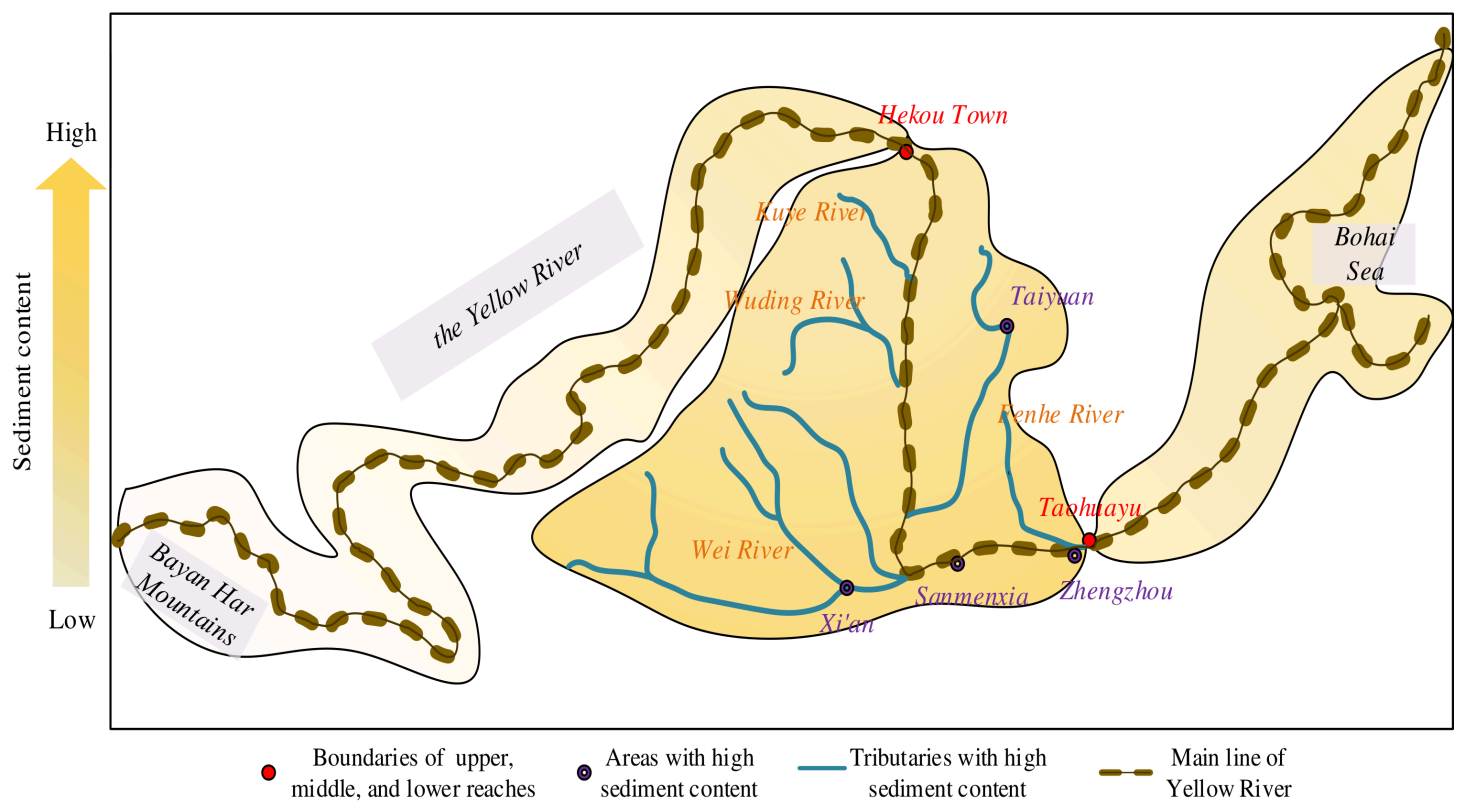
Spatial distribution of sediment content throughout the Yellow River Basin [57,58,59].

The Yellow River traverses diverse geological and climatic zones, creating distinct sedimentary environments across its various reaches [56]. This spatial variability results in regional variation in particle-size distribution [12], mineralogy [5], clay content [45], and chemical composition [50]. Figure 3 illustrates the spatial distribution of sediment content throughout the Yellow River Basin. The map identifies Hekou Town and Taohuayu as the critical boundaries separating the upper, middle, and lower reaches. The highest sediment content is found in the middle reaches and the major tributaries, including the Kuye, Wuding, and Wei Rivers [57,58,59]. These regional differences affect rheological behavior, mechanical properties, and durability, making material performance more difficult to predict across studies and applications [11,44]. Among them, variation in particle-size distribution across the upper, middle, and lower reaches is particularly significant, as summarized in Table 1 [44,60,61,62,63].

## 3. Engineering Applications

Because Yellow River silt and Yellow River sand are used in different ways across construction-material systems, their engineering effects are discussed below according to application mode and performance requirement.

### 3.1. Concrete

#### 3.1.1. Conventional Concrete

Yellow River sand particles are generally rounded and smooth, which reduces inter-particle friction in fresh concrete during mixing and flow. In addition, a considerable proportion of small sand particles increases the specific surface area and requires more paste for adequate coating [35]. When the sand dosage is high, the available paste volume becomes insufficient to meet the elevated demand. Consequently, insufficient paste reduces the cohesiveness and water retention of the fresh mixture, increasing the risk of segregation and bleeding [64].

**Figure 4:**
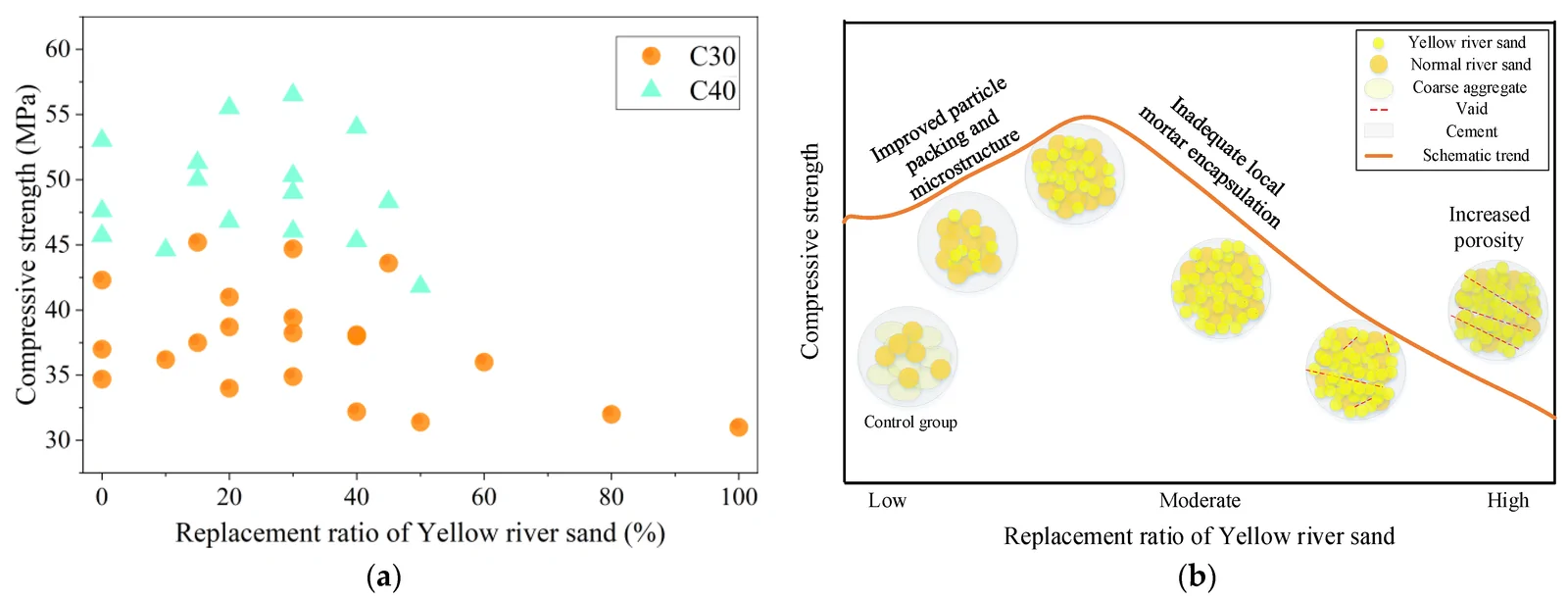
Compressive strength of Yellow River sand concrete: (**a**) Compressive strength data for Yellow River sand concrete from different studies [65,66,67,68,69,70,71]; (**b**) schematic trend of compressive strength evolution with Yellow River sand content.

Figure 4a presents the test data of Yellow River sand concrete collected from different studies [65,66,67,68,69,70,71]. Although the existing research results are discrete, the compressive strength of concrete generally exhibits a non-linear dependence on the replacement ratio of Yellow River sand (defined as the mass percentage of conventional fine aggregate replaced by Yellow River sand). As shown in Figure 4b, compressive strength increases at moderate replacement levels and then decreases when the replacement ratio becomes too high. This trend mainly reflects the balance between the positive and negative effects of Yellow River sand in concrete. At suitable replacement levels, Yellow River sand can refine fine-aggregate grading and fill part of the void space, thereby contributing to a denser internal structure [16]. At excessive replacement levels, the increased specific surface area raises the paste demand, and insufficient paste coverage around the aggregates may lead to internal defects and strength loss [40]. Low- and medium-strength concretes usually show greater tolerance to Yellow River sand replacement, whereas high-strength concretes are more sensitive because of their lower water-to-binder ratio and stricter requirements for mixture compactness. Therefore, replacement ratio alone cannot account for the mechanical performance of Yellow River sand concrete without considering mixture design and sand characteristics. Table 2 summarizes mixture designs, curing conditions, and performance of Yellow River sand concretes [65,66,67,68,69,70,71]. Because the mixture designs differ among the cited studies, the performance values in Table 2 should not be directly compared across studies. At 15% replacement, both C30 and C40 concrete in References [65,66] achieve their highest compressive strengths. In References [69,70], the corresponding levels are 10% for C30 and 30% for C40, with only limited variation in the C30 series. For both grades in Reference [71], the highest values are obtained at 30% replacement. By contrast, Reference [67] shows a continuous decrease in compressive strength as replacement increases from 20% to 100%. Overall, the highest compressive strengths in most series are obtained at replacement levels between 10% and 30%.

Beyond mechanical performance, suitable dosages of Yellow River sand can also improve concrete durability. This effect is generally attributed to a denser internal structure and reduced internal moisture movement, which help limit freeze–thaw damage and slow CO_2_ penetration [66,68]. In addition, the fine particles tend to improve the water retention capacity of the mixture and thereby reduce early-age drying shrinkage [65]. However, when the sand content becomes too high, paste shortage, segregation, and internal defects may offset these benefits.

Unlike Yellow River sand, Yellow River silt contains a much higher proportion of silt and clay particles. This feature can improve the cohesion of fresh concrete and reduce bleeding and segregation, which is beneficial for plastic concrete and low-permeability concrete [72,73]. These fine particles also increase water demand and may reduce admixture efficiency, making strength development more sensitive to silt content. Accordingly, silt content must be carefully controlled in mixture design.

#### 3.1.2. Concrete Incorporating Multiple Solid Wastes

In concrete containing multiple solid wastes, Yellow River silt is commonly used rather than processed Yellow River sand. Because of its fine particle size and high silt content, Yellow River silt can improve the rheology and formability of fresh concrete when used together with industrial solid wastes and recycled materials [16,18,74]. This role is particularly valuable in multi-waste concrete systems, where Yellow River silt helps compensate for discontinuous grading, uneven paste distribution, and the tendency toward high drying shrinkage.

In red-mud and geopolymer concrete, Yellow River silt can bridge the voids between coarse solid-waste particles and improve mixture compactness. This contribution helps support stable strength development within a limited content range [75]. However, this beneficial effect is confined to a limited content range. At excessive silt contents, the abundant fine particles increase water demand and mixture viscosity, thereby impairing both casting quality and hardened performance.

When fly ash and slag are used in concrete, Yellow River silt can mitigate the poor workability and high drying shrinkage often associated with these solid waste materials [75]. Silt helps distribute paste more uniformly among aggregates, which improves early-age workability and leads to more consistent drying-shrinkage behavior. In foamed concrete made with fly ash, silt refines the pore structure and results in a more uniform pore size distribution [18]. This improves thermal insulation while maintaining adequate strength.

In concrete made with recycled aggregates, the fine particles in the Yellow River silt strengthen the paste attachment to the aggregate surface and enable a complete paste coating around the aggregates. This reduces slurry loss during casting and improves forming stability. These findings further suggest that pretreated Yellow River silt can improve not only fresh-state stability but also resistance to erosion under flowing-water conditions [76].

#### 3.1.3. Concrete with Special Properties

In steel fiber-reinforced self-compacting concrete, Yellow River sand can fill the gaps between natural sand and steel fibers, shifting the aggregate grading curve closer to standard zones and ensuring uniform fiber dispersion under high fluidity [77]. When used in zero-slump concrete, the sand absorbs part of the free water and promotes a more uniform moisture distribution in the mixture. The concrete maintains a low slump with limited segregation after mixing, and the vibration compaction produces a dense internal packing [78]. For autoclaved aerated concrete, Yellow River sand can disperse uniformly in the slurry after grinding. This more uniform dispersion contributes to a more homogeneous pore structure and lower pore connectivity [79].

In plastic and low-permeability concretes, the fine particles of Yellow River silt fill the voids within the ITZ. The minor clay fraction in the silt also increases mixture cohesiveness and reduces bleeding. This microstructural densification improves resistance to water penetration and helps maintain the deformation compatibility required in hydraulic and underground works [72,73]. For high-performance concrete, Yellow River sand refines the grading of fine aggregates and allows the mixture to retain high flowability while maintaining strength [35,78]. In activated systems containing slag, Yellow River silt can replace part of the conventional binder [75]. This may reduce cement use, although mechanical performance depends on binder composition and activation conditions. Taken together, these findings indicate that the roles of Yellow River silt and sand in special concretes extend beyond simple material substitution and increasingly involve targeted regulation of fresh-state behavior, pore structure, and sustainability performance [77,78].

### 3.2. Cementitious Materials

Unlike conventional concrete, cementitious materials do not contain coarse aggregates and often use Yellow River sand as the primary fine aggregate [52,79,80,81]. However, the direct use of Yellow River silt in such materials is limited. The ultra-fine particle size and high clay content of silt increase water demand and admixture adsorption, which deteriorates the workability of the fresh mixtures [82]. Furthermore, in engineered cementitious composites (ECC), silt-sized particles create a dense cementitious matrix with high interfacial bond strength [83,84]. Under these conditions, fibers are more likely to rupture than to undergo stable pull-out, which suppresses the characteristic strain-hardening response [85,86]. This is one reason why the direct use of Yellow River silt remains more limited than that of Yellow River sand in cementitious materials.

#### 3.2.1. ECC

ECC has attracted extensive attention because of its high ductility, multiple cracking, and excellent damage tolerance [87,88]. Its performance depends on matrix composition, sand characteristics, and fiber bridging [89,90]. Therefore, the effect of Yellow River sand cannot be assessed by compressive strength alone, but should be examined in relation to ductility, crack control, interface behavior, and durability. The studies reviewed here on the use of Yellow River sediment in ECC were conducted by two independent research groups. Most of these studies came from one group. Although they examine different aspects of ECC performance, broader independent verification is still needed before the findings can be generalized.

Replacing natural sand with Yellow River sand changes the balance between strength and ductility in ECC. Studies on ECC incorporating Yellow River sand show that such replacement affects compressive and flexural strength, while ductility and crack behavior should also be considered in performance evaluation [21,52,91]. Zhang et al. compared ECC prepared with quartz sand and Yellow River sand. They found that complete replacement caused only small reductions in compressive and flexural strengths, whereas tensile response and bending deformation improved [52]. This comparison suggests that Yellow River sand affects ECC through both strength retention and deformation capacity. For 3D-printed ECC, the use of Yellow River sand is further linked with printability, fiber orientation, and anisotropic mechanical response, rather than strength alone [91,92]. From an application perspective, this difference in performance assessment partly explains the different acceptable replacement levels of Yellow River sand in ECC and conventional concrete, since ductility and crack control must be considered in addition to strength.

At the fiber–matrix interface, fine particles in Yellow River sand can improve the local bonding environment around fibers. This improvement helps establish a more stable and uniform bond between the fibers and the cementitious matrix [91,92]. The rounded morphology of sand grains also reduces internal stress concentrations, which facilitates a consistent slip-hardening response during fiber pull-out and redistributes stress along crack initiation and propagation paths. By balancing frictional resistance and chemical bonding, these interfacial optimizations ensure that the energy required for the initiation of multiple cracks remains lower than that required for crack propagation [21,87,93,94].

Beyond mechanical ductility, Yellow River sand influences the durability of ECC, particularly its resistance to freeze–thaw cycles [19,21]. Fine particles refine the pore structure by reducing the most probable pore size and porosity, which mitigates internal damage from ice expansion [21,95,96]. After 300 freeze–thaw cycles, the relative dynamic elastic modulus of ECC exceeds 96.2% [21]. When applied to reinforce frost-damaged concrete, the ECC with Yellow River sand maintains a splitting tensile strength retention rate above 57% after 150 cycles [19,97].

Curing conditions determine the balance between strength development and ductility in Yellow River sand ECC. Standard curing leads to steady strength gain, with the compressive strength at 90 days increasing by 23.9% compared to the 28-day strength. While steam curing accelerates early-age strength, it negatively affects the ultimate tensile strain. In contrast, natural curing promotes stable interfacial bonding, maintaining an ultimate tensile strain above 4% after 90 days [52]. Therefore, the selection of a curing regime depends on the mechanical requirements prioritized for the intended application [98].

One study evaluated a low-cost ECC containing Yellow River silt and locally produced polyvinyl alcohol fibers. Compared with the reference ECC mixture used in that study, energy consumption, carbon dioxide emissions, and estimated material cost were reduced by 15%, 29%, and 88%, respectively [20]. The cost estimate treated Yellow River silt as cost-free and excluded transportation.

#### 3.2.2. Cement Mortar

Yellow River sand can serve as a fine aggregate in cement mortar. Its relatively high mud content can reduce the mechanical strength of the mortar. The incorporation of silica fume and basalt fibers offsets this strength loss, enabling the direct use of unwashed sand and eliminating the need for washing [80]. Furthermore, a recycled mortar containing waste ceramic aggregates and Yellow River sand exhibits higher flexural strength and a higher brittleness coefficient than natural-sand mortar [99].

The rounded particles of Yellow River sand reduce inter-particle friction and improve the flowability of cement mortar [100]. This characteristic meets the rheological and printability requirements for 3D-printed mortar. Experimental results show that the compressive strengths of 3D-printed specimens in the X, Y, and Z directions are lower than those of traditional cast specimens [81,101], although basalt fibers can mitigate this strength reduction. These findings suggest that the main advantage of Yellow River sand in cement mortar lies in improving flowability and printability, whereas its effect on strength varies with mud content, fiber modification, and supplementary materials such as silica fume.

### 3.3. Subgrade Fills

Yellow River silt possesses a well-graded particle distribution, which makes it suitable for highway subgrade fills [102,103]. Under static conditions, the compacted silt exhibits an internal friction angle ranging from 22.6° to 38.1° [103,104]. Under dynamic loading, its dynamic shear modulus and damping ratio follow the Hardin hyperbolic model, demonstrating mechanical behaviors intermediate between those of silty and sandy soils [105,106,107]. Furthermore, long-term cyclic loading tests establish an allowable stress ratio between 0.540 and 0.893 [108,109]. To further enhance the bearing capacity, lime and fly ash are incorporated as stabilizers. When the fly ash dosage exceeds 40%, the resilient modulus of the stabilized silt is greater than 70 MPa, satisfying the mechanical requirements for high-grade highway subgrades [110]. These results show that Yellow River silt can be used as a stabilized subgrade fill material, but its suitability depends on compaction conditions and stabilizer dosage. In this application, engineering performance is evaluated mainly by bearing capacity and deformation stability under static or cyclic loading.

### 3.4. Other Building Products

The use of Yellow River silt in building products mainly follows two routes: (1) high-temperature or hydrothermal consolidation and (2) ambient chemical activation.

Products such as fired bricks, autoclaved bricks, and lightweight ceramsite rely on fine particles for structural formation under high-temperature or hydrothermal conditions. Consequently, Yellow River silt acts as a weakly reactive component in these products. During the firing process, the silt can be synergistically combined with industrial solid wastes (such as red mud) to produce composite fired bricks [111,112]. Under hydrothermal conditions, autoclaved bricks incorporating Yellow River silt achieve a compressive strength of up to 38.76 MPa [113]. Through suitable sintering processes, Yellow River silt can also be converted into lightweight ceramsite for use as structural aggregates [114,115].

Conversely, products formed at ambient temperatures rely heavily on chemical activation to achieve structural integrity. Because Yellow River silt has inadequate mechanical strength, acid-base activators are used in the manufacture of unburned bricks. This activation promotes chemical reactions involving Yellow River silt, refines the pore structure, and improves both compressive strength and frost resistance [75,115,116,117,118,119]. Similarly, in the production of artificial flood-control stones, a composite alkali activator comprising Ca(OH)_2_ and NaOH is used to promote chemical reactions involving Yellow River silt and thereby enhance the compressive strength of the blocks [23,120]. Across both mechanisms, Yellow River sand primarily functions as an inert structural filler due to its larger particle size and limited reactivity [79,121].

Across these applications, Yellow River sediment does not play a uniform engineering role. Yellow River sand mainly serves as an aggregate, whereas Yellow River silt more often acts as a filler or weakly reactive component. This distinction helps explain their different responses among construction materials. Moreover, regional variability can modify these responses even within the same application. Sediments from different reaches vary in grading, clay mineral content, and chemical composition. Under the same mixture design, these variations may alter water demand, admixture adsorption, workability, strength development, and durability. Accordingly, reported application ranges should be treated as reference values rather than fixed limits for all Yellow River sediments.

## 4. Physicochemical Mechanisms and Microstructural Evolution

### 4.1. Physical Packing

The particle characteristics of Yellow River sediment help explain its physical role in construction materials [35,52,122]. Yellow River sand has rounded grains and relatively stable grading, which can reduce friction among particles and improve aggregate arrangement [19,21]. Yellow River silt is much finer and has a larger specific surface area, allowing it to occupy spaces among coarser particles and adjust local density [119]. Therefore, this physical effect should be examined together with evidence from interfacial morphology and pore structure.

Microstructural evidence helps clarify the local packing effect. For ECC prepared with Yellow River sand, scanning electron microscopy (SEM) images in the study by Zhang et al. showed that curing condition affected C-S-H morphology, hydration product density, fiber surface coverage, and gaps near the fiber–matrix interface [52]. These features point to changes in local matrix continuity and fiber–matrix interaction, supporting the discussion of microvoid filling and ITZ densification in cementitious systems containing Yellow River sediment.

**Figure 5:**
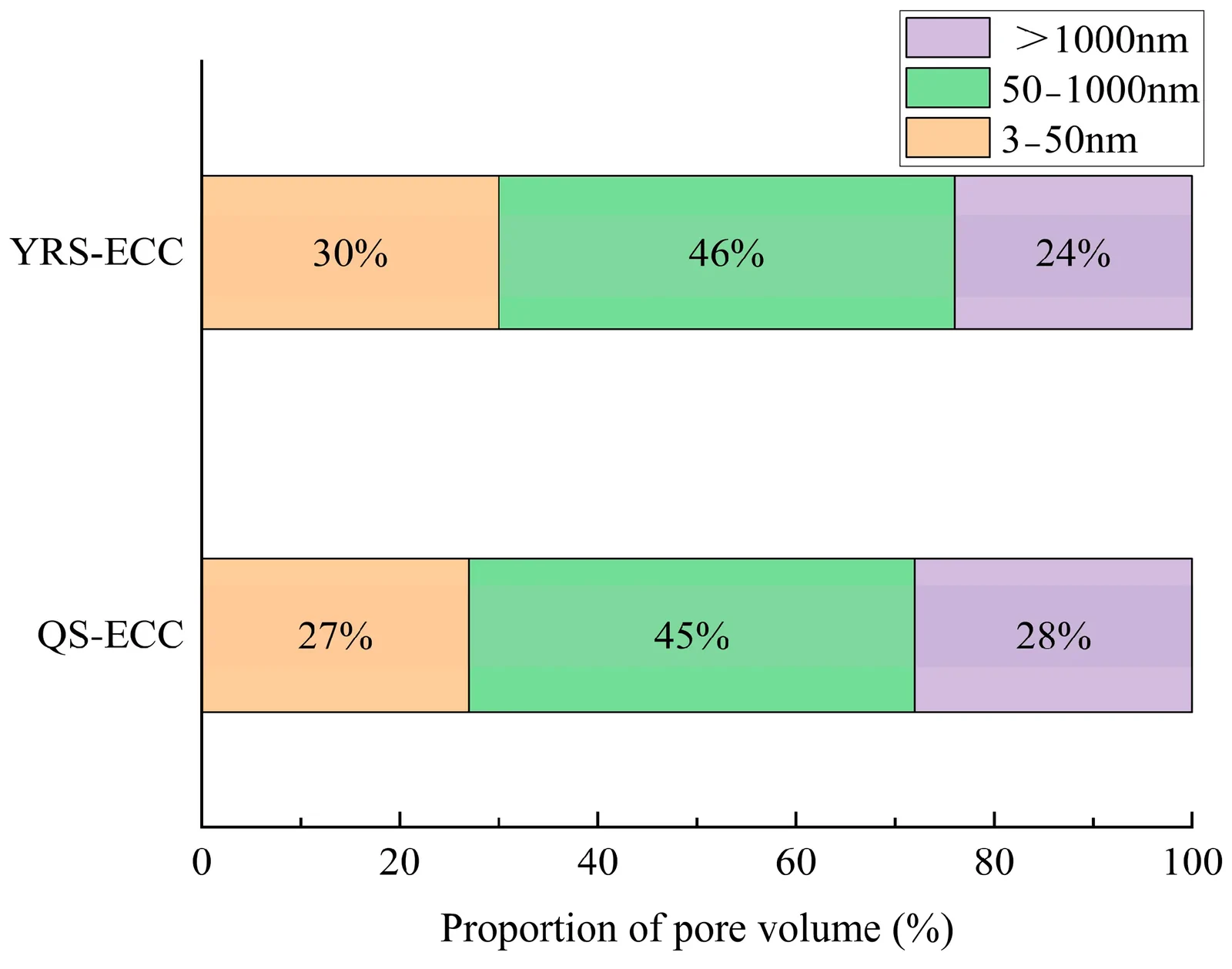
Pore volume fractions in different pore size ranges for ECC prepared with Yellow River sand (YRS-ECC) and with quartz sand (QS-ECC). Reproduced from Tan et al. [19] under the terms of the CC BY 4.0 license.

**Figure 6:**
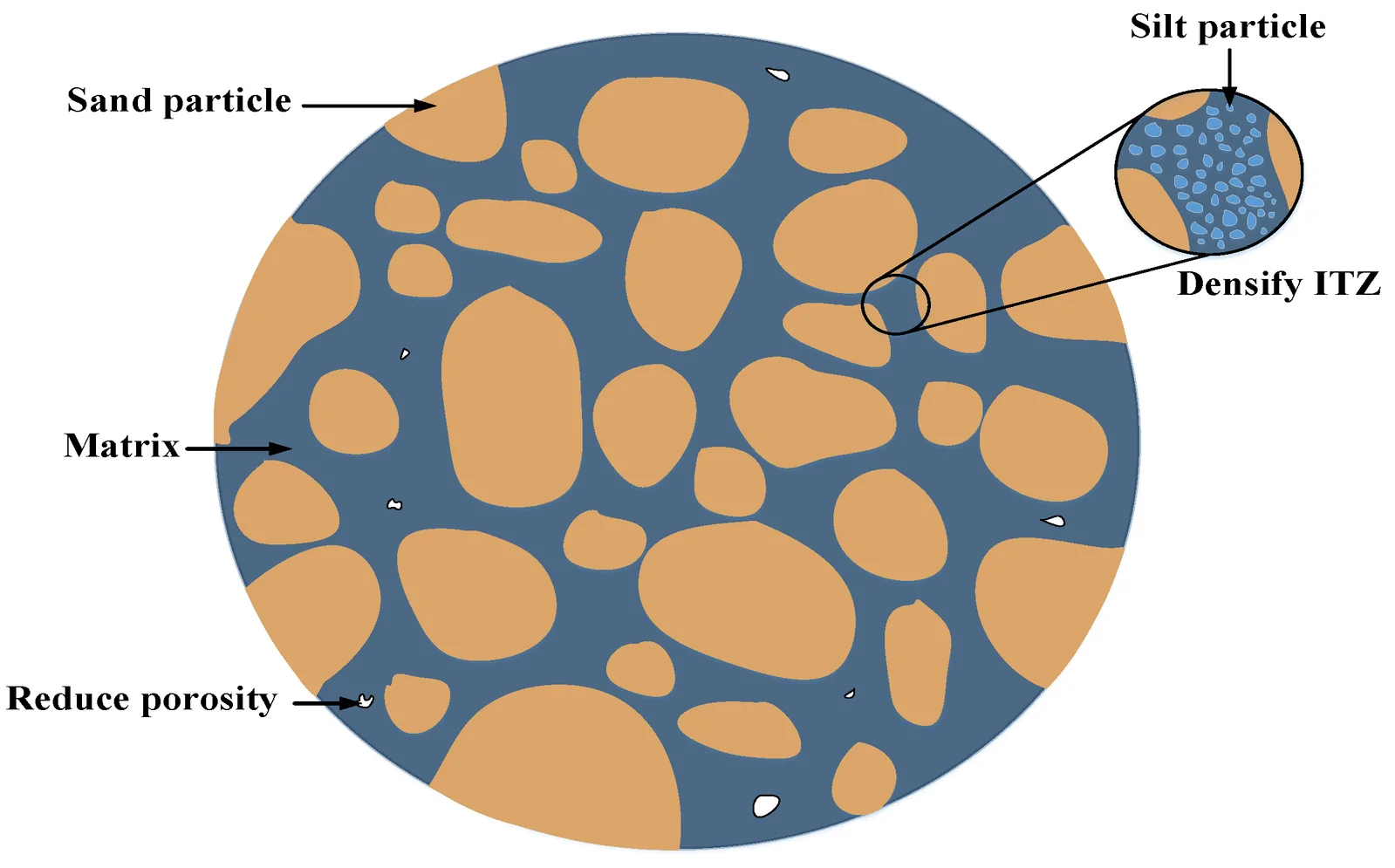
Schematic illustration of sediment packing effects.

At the pore scale, mercury intrusion porosimetry (MIP) provides quantitative evidence for changes in pore structure. Tan et al. [19] compared the pore structures of ECC prepared with Yellow River sand and quartz sand. A lower total porosity was observed in the former mixture. Figure 5 further shows a smaller proportion of pores above 1000 nm in this mixture. Zhang et al. reported that, for ECC prepared with Yellow River sand, curing condition changed porosity and average pore size; specimens subjected to steam curing had the smallest average pore size and a higher proportion of pores below 50 nm [52]. In slag–Yellow River sediment geopolymers, MIP results showed that suitable mineral admixtures increased gel pore content and reduced the proportion of larger pores during early freeze–thaw exposure [123]. These results provide specific evidence for pore refinement through changes in pore size distribution. Figure 6 presents a schematic illustration of sediment packing effects, including local compactness improvement, ITZ modification, and pore structure refinement.

### 4.2. Chemical Reactivity

Chemical reactivity in activated composite binders containing Yellow River silt is mainly reflected in gel formation [25,120]. Under suitable activation conditions, the gels can fill pores, densify the matrix, and contribute to strength development [118]. Yu et al. used X-ray diffraction and thermogravimetry to confirm the presence of C-S-H and C-A-S-H gels. These mixtures contained Yellow River sediment, fly ash, and cement and were activated with NaOH and water glass [117]. Alkali equivalent and water glass modulus also affected the reaction process and product distribution in slag–Yellow River sediment binders [118]. These results show that activator composition and calcium supplied by cement, slag, or Ca(OH)_2_ are integral to the observed gel formation. The contribution of Yellow River silt itself has not been isolated.

The large specific surface area of Yellow River silt provides abundant adsorption sites [124], while clay minerals in the silt adsorb free water and water-reducing agents [45,125]. This adsorption lowers the effective water content and weakens the dispersing action of water-reducing agents, thereby reducing mixture flowability and slowing hydration [121]. Notably, evidence from other river sediments indicates that soluble humic substances can delay cement setting, reduce early-age strength, and increase paste flowability [126]. The required dosage of water-reducing agents may therefore vary with sediment composition, although this has not been quantified for Yellow River sediment [127].

**Figure 7:**
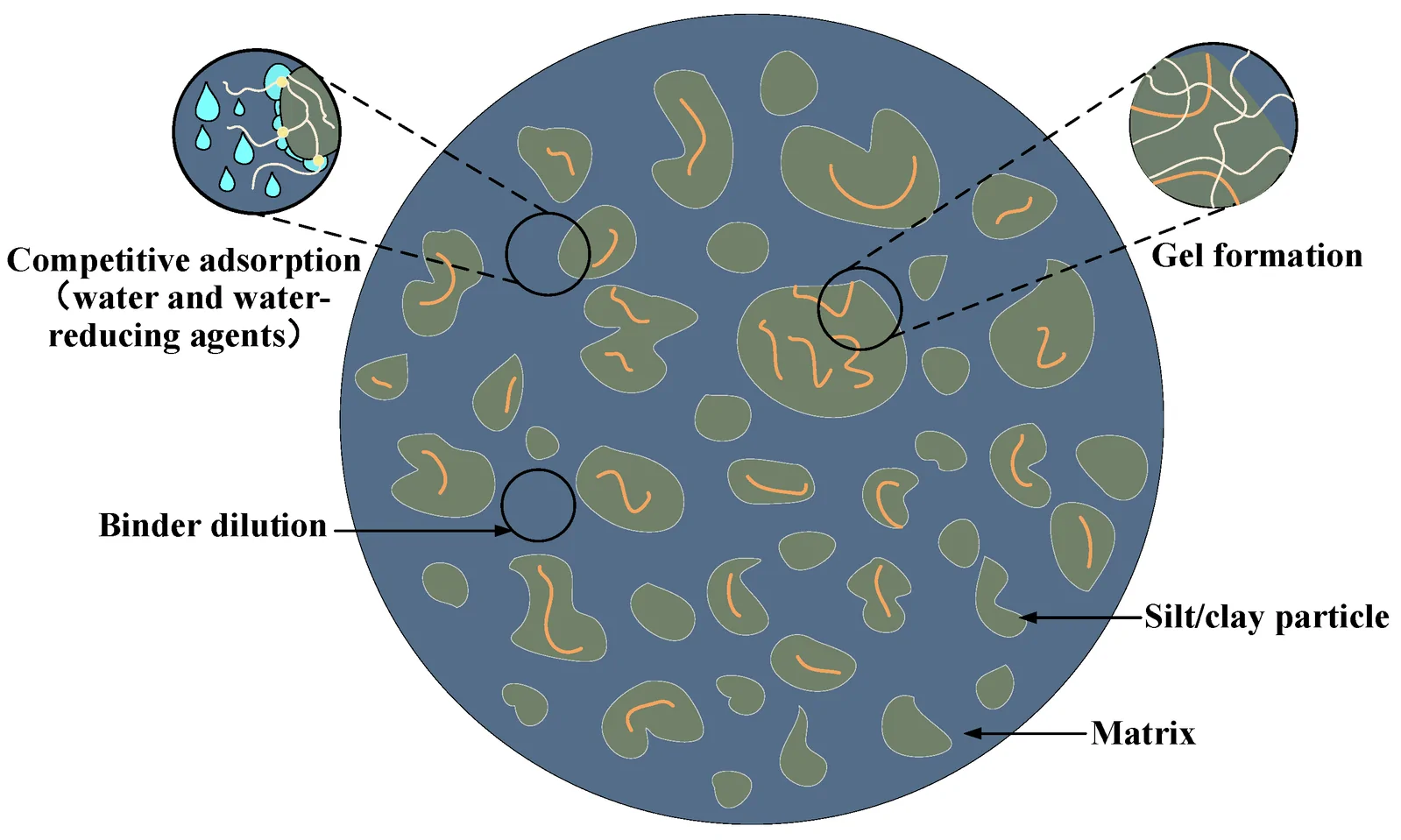
Schematic illustration of chemical contributions of Yellow River silt.

When Yellow River silt replaces part of the cementitious binder, its relatively low reactivity may produce a dilution effect that reduces the degree of hydration and hinders sustained strength development [18,35,121,122]. Figure 7 presents a schematic illustration of chemical contributions of Yellow River silt.

### 4.3. Coupling Mechanism

The application-dependent behavior of Yellow River silt and Yellow River sand is governed by the relative contribution of physical packing and chemical reactivity rather than by either mechanism alone [25,39,118,128]. In some material systems, performance is improved mainly through packing-related effects, such as grading optimization, microstructural densification, and interfacial refinement [39,128]. In others, chemical reactivity plays a more prominent role because gel formation, competitive adsorption, and binder dilution increasingly affect strength development, workability, or durability [118]. Therefore, the same sediment may improve material performance in one application but reduce it in another [123,129].

This shift in relative contribution is controlled mainly by sediment form, sediment content, and activation conditions. Sediment form controls particle-size characteristics and reactive-component availability [25,128], while sediment content governs the balance between positive and negative effects [18,35]. Activation conditions affect chemical reactions in materials containing Yellow River silt [25,117]. Accordingly, the governing mechanism in each material system depends on the balance between these two contributions. Table 3 summarizes the application-specific roles and dominant mechanisms of Yellow River silt and sand in different construction materials.

## 5. Current Challenges and Research Needs

### 5.1. Predictive Models of Material Performance

Reliable prediction of material performance remains difficult for construction materials containing Yellow River sediment. Performance observed for one sediment source or material system cannot be directly transferred to another [18]. Yellow River sediments vary in particle-size distribution, mineral composition, clay content, and specific surface area, and their engineering roles and governing mechanisms also change with material system and mixture design. As a result, current mix design still relies heavily on empirical trials, and standardized engineering use across different sediment sources and applications remains difficult.

Addressing this limitation requires predictive models for specific material systems, supported by cross-regional databases. Candidate sediment descriptors include particle-size distribution, particle shape, specific surface area, mineral and chemical composition, clay content, and water absorption. Depending on the target property and application, appropriate descriptors can be combined with key mixture and processing variables, such as sediment content, replacement mode, water-to-binder ratio, binder or activator composition, pretreatment, and curing conditions. The selected variables can serve as input parameters for predicting rheological behavior, mechanical properties, or durability performance.

### 5.2. Effective Sediment Content Ranges

Determining effective sediment content ranges remains a key challenge for construction materials using Yellow River silt or sand. These ranges are not universal; they vary with sediment form, material system, replacement mode, mixture design, and curing or activation condition. For Yellow River sand, the main constraints are grading, paste demand, workability, and strength retention. For Yellow River silt, these ranges are more closely related to water demand, clay mineral adsorption, activator demand, and dilution of reactive binders.

A clearer basis for content determination is therefore needed. Instead of treating the best content in each mixture as an isolated value, studies should relate sediment characteristics to the controlling performance index and examine a series of sediment contents. The effective range is where the target performance is maintained or improved. The upper limit is where further sediment addition causes performance decline [131]. This would make content ranges more comparable across sediment sources and material systems.

### 5.3. Low-Carbon Activation

Current activation of Yellow River silt still relies mainly on high-temperature calcination or strong alkaline treatment. This reliance stems from the relative stability of its mineral phases, which limits the effectiveness of mild activation [132,133,134]. As a result, methods that substantially enhance reactivity also increase energy consumption, carbon emissions, and processing cost, thereby weakening the sustainability advantage of Yellow River silt utilization. Future research should focus on activation strategies that can improve reactivity under milder conditions. Promising approaches include low-energy mechanochemical treatment and synergistic composite activators [135,136,137].

### 5.4. Long-Term Durability

Long-term durability is still not well established for construction materials using Yellow River silt or sand. The available evidence is mostly drawn from several systems, including ECC mixtures incorporating Yellow River sand and geopolymers incorporating Yellow River sediment [21,39,123]. These studies address frost resistance, drying shrinkage, impermeability, and pore evolution, but they use different binders, exposure conditions, and test indices. Their conclusions remain confined to the tested systems and cannot be directly extended to service conditions.

Future work should therefore focus on deterioration processes during service. Studies on dredged sediment concrete, alkali-activated binders, and marine concrete show that ingress of harmful media, chemical deterioration, and repeated environmental actions are key causes of durability loss [138,139,140]. For Yellow River silt and sand, these processes should be evaluated together with strength retention, dimensional stability, impermeability, and service life assessment.

A further issue is whether these findings can be extended beyond the Yellow River Basin. The engineering roles and mechanisms discussed in this review may also apply to sediments from other river basins with comparable particle and mineral characteristics [8,9,25,26]. The applicable content ranges and performance outcomes must still be verified for sediments from different sources. Desert sand may also be used as a fine aggregate. It often has rounded particles, but this similarity in particle shape does not make it equivalent to Yellow River sand. Differences in grading and particle size can affect workability and mechanical performance [141].

## 6. Conclusions

This review synthesized the physicochemical properties of Yellow River silt and Yellow River sand, their engineering applications, and the mechanisms governing material performance in different construction-material systems. Based on this analysis, the following conclusions can be drawn:

(1) Although Yellow River silt and Yellow River sand originate from the same sedimentary source, they cannot be regarded as equivalent constituents in construction materials. Yellow River sand is mainly associated with concrete, ECC, and cement mortar, whereas Yellow River silt is more commonly involved in subgrade fills, multi-waste concretes, and processed building products.
(2) The beneficial effects of Yellow River silt and Yellow River sand occur only within limited content ranges. These ranges are not universal and depend on sediment form, mixture design, processing condition, and target performance.
(3) Whether Yellow River sediment improves or compromises material performance depends on the relative contributions of physical packing and chemical reactivity. These contributions are affected by sediment form and content, as well as by the binder environment and processing conditions. The evidence for chemical reactivity comes mainly from systems containing Yellow River silt, but the contribution of the silt itself has not been isolated.
(4) Regional variability is a major obstacle to the standardized engineering use of Yellow River sediment. Sediments from different origins and depositional settings exhibit distinct material characteristics and engineering performance, which makes it difficult to establish design criteria applicable across regions. Future research should focus on predictive models of material performance, effective sediment content ranges, low-carbon activation methods, and long-term durability under complex service conditions.

## Figures and Tables

**Table 1:** Particle-size distribution of Yellow River sediment (%) [44,60,61,62,63].

Geographical Location	Cumulative Screening Rate		
	<0.002 mm	0.002–0.063 mm	>0.063 mm
Upper reach	0.84–15.90	23.06–67.80	6.20–66.10
Middle reach	10.30–15.70	44.80–51.30	34.30–44.90
Lower reach	0.93–14.24	29.67–71.46	14.31–75.61

**Table 2:** Comparison of mixture designs, curing conditions, and performance of Yellow River sand concrete.

Source	Concrete Grade	Binder Composition	Yellow River Sand Replacement Ratio (%)	Water-to-Binder Ratio	Mud Content of Yellow River Sand (%)	Curing Condition	28-Day Compressive Strength (MPa)	Durability
References [65,66]	C30	Cement + fly ash + slag powder	0	0.48	3.8	Standard curing	42.3	15% replacement minimizes early-age shrinkage [65] and optimizes carbonation and chloride resistance [66]; 30% replacement maximizes freeze–thaw resistance [66].
			15				45.2	
			30				44.7	
			45				43.6	
	C40		0	0.41			47.6	
			15				51.3	
			30				50.3	
			45				48.3	
Reference [67]	C30	Cement + fly ash	20	0.46	0.6	Standard curing	41	/
			40	0.46			38	
			50	0.46			36	
			80	0.45			32	
			100	0.45			31	
Reference [68]	C30	Cement + fly ash	30	0.5	1.9	Standard curing	38.2	30% replacement reduces permeability and improves freeze–thaw resistance.
	C40		30	0.42			46.0	
References [69,70]	C30	Cement + fly ash	0	0.5	1.6	Standard curing	34.7	/
			10				36.2	
			20				34.0	
			30				34.9	
			40				32.2	
			50				31.4	
	C40		0	0.42			45.7	
			10				44.6	
			20				46.8	
			30				49.0	
			40				45.3	
			50				41.8	
Reference [71]	C30	Cement + fly ash + slag powder	0	0.47	2.4	Standard curing	37.0	/
			15				37.5	
			20				38.7	
			30				39.4	
			40				38.1	
	C40		0	0.4			53.0	
			15				50.0	
			20				55.5	
			30				56.5	
			40				54.0	

Note: “/” indicates that no relevant information is reported.

**Table 3:** Application-specific roles and dominant mechanisms of Yellow River silt and sand in construction materials.

Application Category	Yellow River Sediment Form	Typical Role	Main Engineering Effect	Key Limiting Factor	Dominant Mechanism
Conventional concrete	sand	Fine-aggregate replacement	Strength and durability improvement [65,66,67,68,69,70]	Sand replacement ratio	Physical packing
	silt	Fine cohesive fraction in plastic and low-permeability concrete	Segregation and bleeding reduction [72,73]	Silt content	
Concrete incorporating multiple solid wastes	silt	Fine filler and rheology modifier	Workability and forming stability improvement [22,74,76]	Silt content	Physical packing
Concrete with special properties	sand	Fine aggregate and grading refinement	Fresh-state stability, pore regularity, and flow retention improvement [64,77,78]	Mixture type and curing condition	Physical packing
	silt	ITZ filler and binder component in activated systems	Water resistance improvement; reduced cement use in activated systems [72,73,75,130]	Silt content and processing condition	Physical packing; chemical reactivity in activated systems
ECC	sand	Fine aggregate	Crack control and ductility improvement [19,21,52]	Sand replacement ratio and curing condition	Physical packing
Cement mortar	sand	Fine aggregate	Flowability improvement [80,100]	Mud content	Physical packing
3D-printing mortar	sand	Fine aggregate	Printability improvement [81,101]	Fiber modification	Physical packing
Subgrade fills	silt	Fill material	Bearing capacity improvement [35,40]	Initial state and stabilizer dosage	Physical packing
Thermally and hydrothermally consolidated building products	silt	Weakly reactive component	Consolidation and strength development [111,112,113,114,115]	Thermal and hydrothermal treatment	Chemical reactivity
Ambient-activated building products	silt	Weakly reactive component	Strength and frost resistance improvement [23,118,119,120]	Type and dosage of activator	Chemical reactivity

Note: Dominant mechanism refers to the mechanism that is more directly associated with the observed engineering outcome in a given application; it does not exclude the concurrent contribution of the other mechanism.

## Data Availability

No new data were created or analyzed in this study. Data sharing is not applicable to this article.
